# Effective Extracorporeal Photopheresis of Patients with Transplantation Induced Acute Intestinal GvHD and Bronchiolitis Obliterans Syndrome

**DOI:** 10.3390/biomedicines10081887

**Published:** 2022-08-04

**Authors:** Robin Reschke, Stephanie Zimmerlich, Christine Döhring, Gerhard Behre, Mirjana Ziemer

**Affiliations:** 1 Department of Pathology, University of Chicago, Chicago, IL 60637, USA; 2 Department of Dermatology, Venereology and Allergology, University Medical Center Leipzig, Philipp-Rosenthal-Str. 23, D-04103 Leipzig, Germany; 3 Medical Department I–Hematology and Cell Therapy, Medical Oncology, Hemophilia, University Medical Center Leipzig, D-04103 Leipzig, Germany; 4 Städtisches Klinikum Dessau, Medizinsiche Hochchule Brandenburg, Klinik für Innere Medizin I, Auenweg 38, D-06847 Dessau, Germany

**Keywords:** extracorporeal photopheresis, acute intestinal graft-versus-host disease, bronchiolitis obliterans syndrome

## Abstract

**Background**: Patients with steroid-refractory intestinal acute graft-versus-host disease (aGvHD) and bronchiolitis obliterans syndrome (BOS) represent a population with a high need for alternative and effective treatment options. **Methods**: We report real-life data from 18 patients treated with extracorporeal photopheresis (ECP). This cohort consisted of nine patients with steroid-refractory intestinal aGvHD and nine patients with BOS. **Results**: We document partial or complete clinical response and reduction of symptoms in half of the patients with intestinal acute GvHD and patients with BOS treated ECP. Responding patients tended to stay on treatment longer. In patients with BOS, stabilization of lung function and forced expiratory volume was observed, whereas, less abdominal pain, less diarrhea, and a reduction of systemic corticosteroids were seen in patients with intestinal acute GvHD. **Conclusions**: ECP might not only abrogate symptoms but also reduce mortality caused by complications from high-dose steroid treatment. Taken together, ECP offers a serious treatment avenue for patients with steroid-refractory intestinal acute GvHD and BOS.

## 1. Introduction

Extracorporeal photopheresis (ECP) is an immunomodulatory treatment procedure based on the combination of leukapheresis with photochemotherapy. Originally developed and approved for the treatment of cutaneous T-cell lymphoma, the use of ECP is constantly expanding for further indications. These include acute graft-versus-host disease (aGvHD) after allogeneic bone marrow or peripheral stem cell transplantation (aHSCT) and bronchiolitis obliterans syndrome (BOS) after lung transplantation [1].

Despite many advances in aHSCT, aGvHD may occur in up to 60% of recipients, even when the donor is a sibling who is identical at the major histocompatibility (HLA) locus [2]. Acute GvHD is characterized by high morbidity and mortality [3]. After skin, the gastrointestinal tract is the second most frequently involved organ [4]. Especially severe aGvHD of the lower gastrointestinal tract is frequently resistant to systemic corticosteroid treatment and the gastrointestinal tract is involved in virtually all fatal cases of aGvHD [5].

The treatment of BOS is also considered a challenge in long-term follow-up after lung transplantation. BOS is a delayed allograft dysfunction with a persistent decline in forced expiratory volume in 1 s (FEV1) not originating from other known and potentially reversible causes and is one of the main factors for morbidity and mortality after lung transplantation [6].

One potential treatment option for these patients is ECP [1]. During ECP, the patient’s leucocytes are collected by apheresis, incubated with the DNA-intercalating and photosensitizing agent 8-methoxypsoralen, exposed to ultraviolet light A (UVA), and returned to the patient. ECP is known to induce cellular apoptosis of leucocytes with secondary immunomodulating effects on dendritic cells, which in sum result in strong anti-inflammatory effects used for treatment and prevention of a number of diseases, including GvHD and solid organ grafts rejections [7]. The aim of this work is to evaluate the efficacy of ECP in patients with steroid-refractory aGvHD of the gastrointestinal tract after aHSCT and in patients with BOS after lung transplantation using real-life data from clinical practice. There is early evidence from a retrospective analysis that ECP in patients with steroid-refractory aGvHD is superior to anticytokine therapy in terms of overall response and survival [8]. Similarly, ECP seems to stabilize lung function in patients with BOS [9]. However, data supporting this treatment concept for these two conditions is still scarce, and investigated cohorts are small. ECP might lead to a decrease in corticosteroid usage and mortality in this high-need patient population.

## 2. Material and Methods

The study included all patients with steroid-refractory intestinal aGvHD after aHSCT who received treatment with ECP between January 2008 and December 2017 (nine patients) and all patients with BOS after lung transplantation who received treatment with ECP between May 2010 and December 2017 (nine patients) in the Department of Dermatology, Venerology and Allergology of the University Medical Center Leipzig. Patients with steroid-refractory intestinal aGvHD were taken care of as inpatients at the special transplant units of the Department of Hematology of the University Medical Center Leipzig. Patients with BOS stayed in hospital only during the days of the ECP procedure. Data were collected retrospectively, using electronic databases of SAP- and COPRA-patient-data-management system as well as HydMedia G5 (Agfa HealthCare) data archive. Response of intestinal aGvHD to ECP was assessed based on clinical data (presence of hematochezia and/or severe abdominal pain requiring medication) and measurement of daily diarrhea volumes and frequency. The volume of diarrhea was documented daily and measured by the content of bedpans or aspirates of intestinal tubes (excluding individual episodes of diarrhea during ECP treatment or defecation attributed to bowel preps for endoscopy procedures). Intestinal aGvHD was staged and graded according to established criteria [10]. Grade 0 is defined as diarrhea < 500 mL/day or frequency < 3/day, grade 1 as diarrhea 500–999 mL/day or frequency 3–4/day, grade 2 as diarrhea 1000–1500 mL/day or frequency 5–7/day, and grade 3 as diarrhea > 1500 mL/day or frequency > 7/day [10]. Grade 4 however, is characterized as severe abdominal pain with or without ileus and bloody stool independent of stool volume [10]. 

The diagnosis of intestinal aGvHD was based on clinical signs after exclusion of other diarrhea-causing diseases and, if available, on results of endoscopy procedures with biopsy and histological examination. Steroid-refractory GvHD was defined as progression within two days or absence of improvement within five to seven days despite parenteral treatment with methylprednisolone at 2 mg/kg body weight/d or equivalent. Immunosuppression with systemic glucocorticosteroids (prednisolone, methylprednisolone, hydrocortisone) has been documented and its dosage used as an indirect indicator for evaluating therapy response. Assessment included the following criteria:

Complete response (CR): statistically significant reduction of the systemic corticosteroid to ≤10 mg/d prednisolone equivalent or discontinuation and no more diarrhea.

Partial response (PR): statistically significant reduction in diarrhea with long-term and statistically significant reduction in systemic corticosteroid (but still more than 10 mg/d prednisolone equivalent).

No significant response (NR): worsening or lack of statistically significant reduction in diarrhea or increase in systemic corticosteroid dose.

Patients after lung transplantation who developed a persistent and progressive decrease in lung function despite systemic immunosuppression and azithromycin were diagnosed with BOS after other known and potentially reversible causes were excluded. The staging of BOS was based on the classification system of the International Society for Heart and Lung Transplantation (ISHLT) [11]. The classification from 2002 describes BOS0 as forced expiratory volume in the first second (FEV_1_) > 90% and forced expiratory flow between 25% and 75% of vital capacity (FEF_25–75_) > 75% of the baseline value, BOS0p as FEV_1_ 81–90% and/or FEF_25−75_ < 75% of the baseline value, BOS1 as FEV_1_ 66–80% of the baseline value, BOS2 as FEV_1_ 51–65% of the baseline value, and BOS3 as FEV_1_ < 50% of the baseline value [11].

FEV1 was measured by spirometry. For each patient, FEV1 values–beginning from the best FEV1 value after lung transplantation up to the FEV1 value at the end of treatment with ECP–were documented in a point diagram. Rapidity of the FEV1 decrease (l/s/month) in the period of six months prior to ECP was compared to the FEV1 course during the period of ECP treatment. Response to therapy was determined on the following criteria:

Significant response: Statistically significant reduction of rapidity of FEV1 decrease (l/s/month) after the beginning of ECP treatment.

Clinically objectifiable but statistically not significant response: reduction of rapidity of FEV1 decrease (l/s/month) during ECP treatment, which, however, is not statistically significant.

No response: neither clinically objectifiable nor statistically significant reduction of rapidity of FEV1 decrease (l/s/month) during ECP treatment.

ECP was performed using either the UVAR XTS^®^ or the CELLEX^®^ closed-system photopheresis from Therakos (Mallinckrodt Pharmaceuticals, (Therakos (UK) Ltd., New Jersey, USA). In patients with intestinal aGvHD, ECP was performed twice a week on non-consecutive treatment days until the maximum therapeutic response. In patients with BOS, ECP was performed every two weeks. One treatment cycle consists of two sequential days. Cycle intervals were expanded only in case of clinical improvement. This study was approved by the University Ethics Committee Leipzig, Germany (338/17-ek).

## 3. Statistics

Data were evaluated with the statistical software SPSS version 24.0. The characteristics of the individual groups were defined by using methods of descriptive statistics. In patients with intestinal aGvHD, the relation was described by using linear regression analysis with the amount of diarrhea and the systemic corticosteroid dose as dependent variables as well as the regression coefficient (r). In patients with BOS, the T-test for independent samples and the Levene test were used to compare the mean values of normally distributed interval-scaled variables. In all tests, significance was assumed for values of *p* < 0.05.

## 4. Results

### 4.1. Acute Intestinal Graft-Versus-Host Disease

Patient characteristics are shown in Table 1.

**Figure 1:**
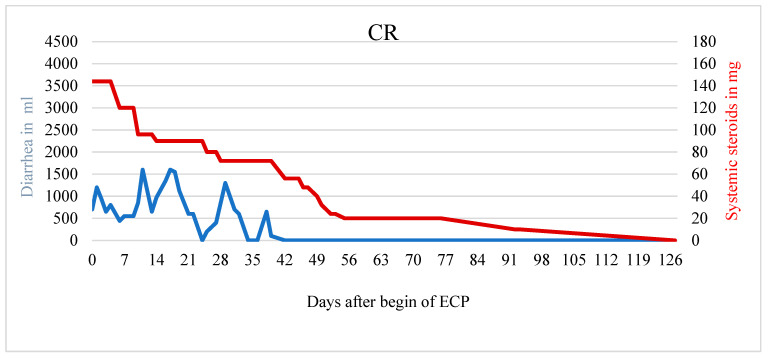
Patient with CR and significantly less diarrhea and usage of systemic steroids. The patient reported a decrease in abdominal pain during ECP.

**Figure 2:**
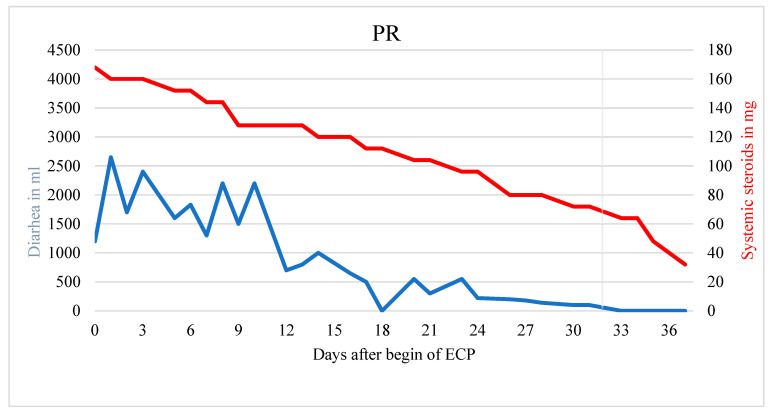
Patient with partial response. Significant reduction of diarrhea and steroid usage during ECP treatment. End of ECP marked with a vertical gray line.

**Figure 3:**
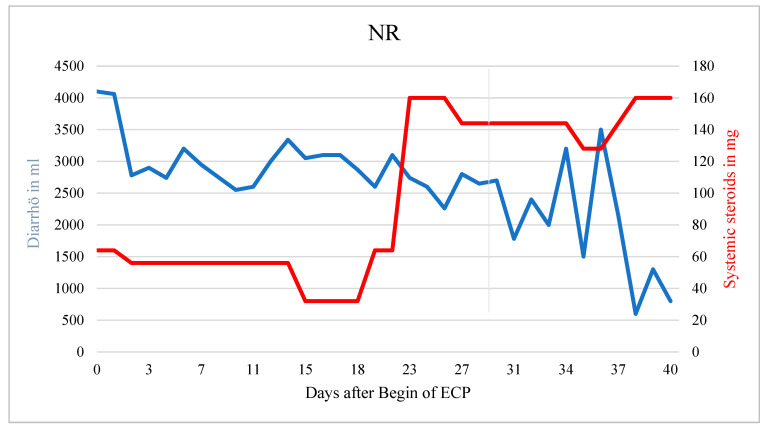
Patient with NR. Corticosteroids had to be increased during ECP due to hematochezia. Diarrhea decreased over time but no significant treatment response was observed in this patient.

A total of five of nine patients showed response to therapy with ECP (two patients complete response, three patients partial response). Exemplary cases of therapy response are demonstrated in Figure 1, Figure 2 and Figure 3. Figure 1 displays a patient with complete response to ECP in terms of both, reduction of diarrhea (r = −12.389; *p* < 0.001) and reduction of systemic corticosteroid (r = −1.368; *p* < 0.001) (Figure 1).

Figure 2 demonstrates a patient with partial response with statistically significant reduction in diarrhea (r = −63.839; *p* < 0.001) as well as of the systemic corticosteroid (r = −3.231; *p* < 0.001), however with a remaining corticosteroid dose of >10 mg prednisolone equivalent (Figure 2).

The patient died from sepsis with multiorgan failure before steroids could be tapered further. In a patient without response neither the systemic corticosteroid dose nor the amount of diarrhea could be reduced (r = 3.311, *p* < 0.001) (Figure 3).

The slightly decreasing amount of diarrhea (r = −43.426; *p* < 0.001) was most likely the result of an increased corticosteroid dose. In general, treatment response seemed not to depend on the clinical stage of aGvHD at the onset of ECP (Table 2).

With regard to response to therapy and the beginning of treatment with ECP, the following distribution is shown: in the two patients with CR, ECP was started 8 and 21 days after diagnosis of steroid-refractory intestinal aGvHD. In the patients with PR, ECP was started at a median of 19 days (range 13–62), while in patients without significant response to therapy, ECP was started at a median of 38 days (range 15–42) after diagnosis. The two patients with CR received 26 and 34 ECP treatments in 128 and 283 days. In the patients with PR, a median of 9 ECP treatments (range 8–25) were carried out in the median treatment period of 32 days (range 24–98). The patients who showed no significant response to therapy received a median of 13 ECP treatments (range 6–37) over a median treatment period of 54 days (range 21–140). In the present study, four patients received fewer than ten ECP treatments because of the worsening of their general condition. In these four patients, there was either no response or only a partial response to therapy. On average, the patients with partial response to fewer than 10 ECP treatments had a slightly higher number of ECP treatments (8.5 vs. 7.5).

Overall, a response to ECP was observed in the first three months. A partial response to ECP treatment achieved a median after 1.07 months (range 0.4–2.2) and a complete response achieved a median after 2.8 months (range 2.6–3). In the two patients with complete response, the systemic corticosteroid was completely discontinued. Apart from one patient, where mycophenolate mofetil could be reduced, non-steroid immunosuppressive medication was not changed. A further four patients with intestinal aGvHD had severe abdominal pain. In both patients with a complete response, there was distinct relief from abdominal pain so pain medication was no longer necessary. ECP also reduced abdominal pain in another two patients, even if there was no significant reduction in diarrhea. Regarding survival, eight of nine patients with intestinal aGvHD died during the study period (89%). The most common clinical cause of death was sepsis with multi-organ failure. A summary of the results is shown in Supplementary Table S1.

### 4.2. Bronchiolitis Obliterans Syndrome

Patient characteristics are shown in Table 3.

**Figure 4:**
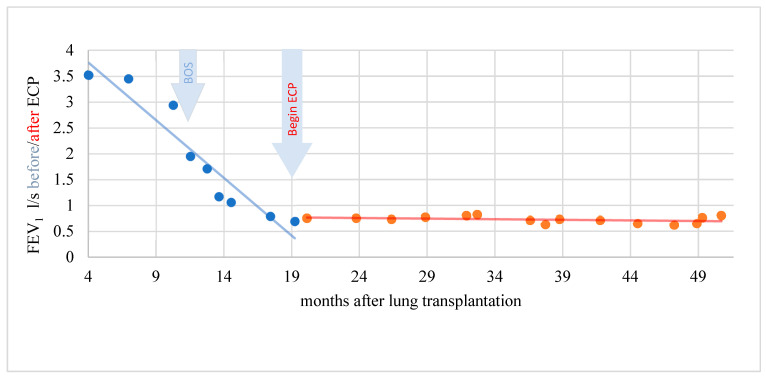
Complete response of a patient with BOS to ECP. Lung function and FEV1 stabilized.

**Figure 5:**
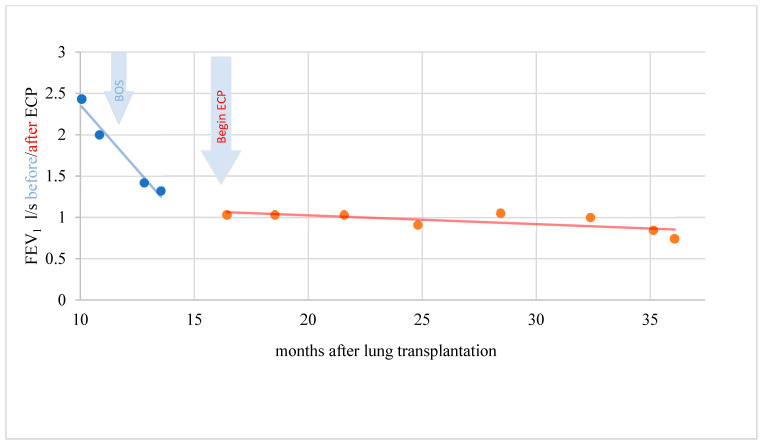
BOS patient with objectifiable but not significant response during ECP.

**Figure 6:**
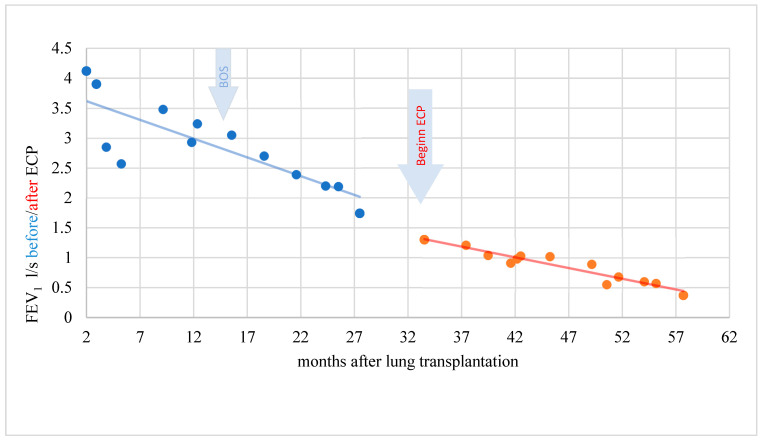
BOS patient without response and decreasing lung function during ECP.

A total of four of nine BOS patients showed response to therapy with ECP (two patients with significant response and two patients with clinically objectifiable but statistically not significant response). Exemplary cases of therapy response are demonstrated in Figure 4, Figure 5 and Figure 6.

In a period of 15 months before the start of ECP, the rapidity of FEV1 reduction was on average–0.229 l/s/month (standard deviation 0.305). A further decline in lung function could be stopped during therapy with ECP and the FEV1 even increased by an average of 0.011 l/s/month (standard deviation 0.049) over a treatment period of 31 months. This difference was statistically significant (t = −2.346; *p* = 0.046). In another patient, the rapidity of FEV1 reduction was distinctly reduced under ECP. The difference, however, was not statistically significant (t = −1.903; *p* = 0.123) and the response was therefore evaluated clinically objectifiable but statistically not significant (Figure 5).

In a patient without response, the rapidity of FEV1 decrease was reduced only minimally under ECP being statistically not significant (t = −0.361; *p* = 0.722) (Figure 6).

The patients’ overall treatment responses depending on the BOS stage are shown in Table 4.

Both patients with BOS stage 1 at the beginning of ECP treatment had either a significant or a clinically objectifiable but statistically not significant response. Three of four patients with BOS stage 3 showed no response to therapy while one patient responded significantly. Patients with significant response to ECP remain on treatment longer than patients with less or missing response. Systemic corticosteroids or other systemic immunosuppression could not be reduced in any patient. Of the nine patients included in the study, two patients died during the study period. The cause of death was most likely sepsis with multiple organ failure and respiratory failure in BOS. One patient’s survival is unknown. Six patients were still alive at the end of the study. Two of these patients were still on ECP therapy. In the remaining four patients, ECP therapy was terminated: but in only one patient ECP was terminated due to long-term stable lung function at the last twelve-week interval after a total of 50 treatment cycles within 72 months. FEV1 remained stable without ECP therapy. In this patient, who had already started ECP therapy in BOS stage 1, there was a significant response to therapy. In the other three patients, ECP was discontinued because of lung re-transplantation, a poor general condition that no longer allowed the long journey to the ECP center or the patient himself refused treatment. Further characteristics are shown in Table 5.

## 5. Discussion

### 5.1. Acute Intestinal Graft-Versus-Host Disease

In 1998 Greinix et al. reported on the positive effect of ECP treatment in patients with aGvHD [12]. Despite the limited number of studies and patients, especially patients with gastrointestinal tract involvement, there is evidence that patients who do not respond to first-line therapy can benefit from treatment with ECP [12,13,14,15]. Generally, in larger international studies the response to ECP therapy appears to be dependent on the severity and the organ manifestation of aGvHD. For organ-specific response, the response of skin was the highest, followed by gut and liver [16].

The results of our study confirm that steroid-refractory intestinal aGvHD is extremely difficult to treat. In our patients–all with progressive acute gut GvHD at the beginning of treatment with ECP–there was no relation between therapeutic response and clinical stage of aGvHD. Therefore, even patients with high-stage aGvHD can significantly respond to therapy. In our study, ECP was started after a median of 21 days (range 8–62) after diagnosis of intestinal aGvHD. The large range results from the fact that in some patients the intestinal aGvHD initially responded to systemic corticosteroids but subsequently worsened and became steroid-refractory. Overall, the majority of ECP-responding patients (CR, PR) started ECP earlier (8–21 days after diagnosis). Thus, starting therapy earlier might be associated with a better response.

In our study, a response to ECP was observed in the first three months. This result supports the statement, that a response to treatment can be expected after only four weeks of therapy and that the patients show a significant improvement in the first three months of treatment [1,17]. The ECP started with two treatments per week on non-consecutive days. This intensive treatment regimen is particularly recommended in patients with intestinal acute or chronic GvHD [15]. Regarding the total number of ECP treatments, four patients in the present study received fewer than 10 ECP treatments. In these, the small number of treatments was not sufficient to achieve a response or to maintain the initial partial response. In the two patients with complete response, ECP was continued over several months with a slow extension of treatment intervals in order to maintain the stability of GvHD. Nevertheless, in none of the patients, additional immunosuppressive medication could be terminated. In other studies, further non-steroid drug immunosuppression could also be reduced or stopped only to a limited extent [12,14]. In addition to improvement of diarrhea and sparing of systemic steroids, in our patients, ECP had also had a beneficial effect on abdominal pain allowing discontinuation of the analgetic medication. Moreover, in these seriously ill and multimorbid patients, with adjustable circulatory problems, no higher-grade side effects of ECP treatment were observed. Overall, the one-year survival rate of the patients included in the study is 10% lower than the 30% described in the literature but confirms the generally poor prognosis of steroid-refractory aGvHD [14]. Treatment of refractory GvHD usually does not result in a patient’s death directly. Leading causes of death are complications caused by steroid-refractory GvHD such as sepsis or multi-organ failure even if there is an on-target treatment response seen in intestinal aGvHD.

### 5.2. Bronchiolitis Obliterans Syndrome

The treatment options for BOS are limited and a lack of control of this disease is the main barrier to better long-term survival after lung transplantation. In the literature, ECP in combination with standard immunosuppressive therapy is the most tolerable and most effective therapy with the highest evidence under salvage therapies (level C, recommendation class IIb) [9]. Overall, studies for ECP in BOS after lung transplantation are limited and the available data are based on expert opinions, studies with small numbers of patients, and retrospective analyses. Nevertheless, several studies showed an improvement or stabilization of FEV1 after the start of ECP and also improved survival rates compared to the control group [9,18,19,20,21]. Unfortunately, for the majority of patients in the present study, we were unable to reduce the decline in lung function with ECP therapy significantly. This result confirms that progressive BOS is very difficult to treat despite existing multimodal therapy. One of the reasons for the therapeutic challenge is the diverse course of the disease. In the present study, the BOS was clinically very heterogeneous with varying degrees of manifestation and either slow worsening in lung function over years or rapid loss of lung function within a few months. Regarding the individual response groups, it is noticeable that five patients who did not respond to therapy were already at an advanced BOS stage > 1 at the beginning of ECP. This indicates a tendency that an advanced BOS stage might be associated with a poorer response to therapy. The prospective study by Jaksch et al., in which 51 patients received ECP in addition to triple drug immunosuppression, azithromycin, and proton pump inhibitors, shows a better treatment response to ECP in the lower BOS stage (*p* = 0.05) [19]. The majority of patients had BOS stage 3 at the start of ECP treatment (68%). In the present study, the majority of patients had stage 3 at the start of ECP, too. Nevertheless, the results show that even with advanced stage 3 disease, therapy success can be achieved in the sense of a significant reduction in the rate of the fall in FEV1 (l/s/month) after the start of ECP therapy. Observational studies suggested that the BOS-related decline in FEV1 was not continuous. According to Lama et al., the decline in FEV1 should be greatest in the first six months and then stabilize [22]. With regard to the treatment response and the start of therapy with ECP, there was no correlation in the present study. Although the patient with the shortest interval between diagnosis and start of ECP treatment responded significantly to ECP, other patients who also started therapy early (≤3 months) did not show a significant response. The optimal start of therapy using ECP has not yet been clearly defined. The decision to start therapy with ECP is made in each individual case by the treating pneumologist. Benden et al. recommend an early start of therapy because developing fibroproliferation of the small respiratory tracts is hardly reversible with ECP [9]. With regard to an early start of therapy, the retrospective study by Isenring et al. showed better long-term survival over a period of five years for patients with BOS after lung transplantation who started ECP therapy in BOS stage 1 [23]. Although patients with BOS stage > 1 were still alive in the present study, the best quality of life without the need for oxygen therapy was shown for patients who started ECP therapy early in BOS stage 1. In most of the studies, ECP therapy was predominantly performed with the UVAR XTS^®^ system. According to Chionis et al., who compared the Therakos UVAR XTS^®^ and CELLEX^®^ systems for the first time in patients with BOS after lung transplantation, the same response rates were found with regard to a reduction in FEV1 decrease and survival [24]. Regarding the duration of treatment, long-term treatment is recommended in response to therapy in order to maintain the clinical response [25]. In the present study, the two patients with significant treatment responses received ECP over several years. However, ECP was continued for as long as possible even in patients who showed no significant response. Despite long-term ECP therapy, none of the patients was able to stop drug immunosuppression.

Overall, the significance is limited in the present study due to the small number of patients. Nevertheless, the data also reflect the results of other collectives. ECP is a treatment option that is comparably well tolerated even for seriously ill patients.

## 6. Conclusions

The present results confirm that an advanced steroid-refractory illness is difficult to treat in both BOS and acute intestinal GvHD but remarkable therapy success can be achieved with ECP. An early start of ECP should be considered and the treatment regimen should be adjusted individually. Independent of the clinical acute intestinal GvHD stage, patients with either partial or complete remissions showed a significant reduction in diarrhea and systemic corticosteroid usage. In addition, a reduction in abdominal pain was observed. Patients with complete remission underwent ECP for a median of 2.8 months and therefore longer than patients with partial remission, indicating that a minimum of treatment cycles might be necessary to achieve a more sustainable response. In analogy to the findings in intestinal aGvHD, we found positive effects of ECP in patients with advanced steroid-refractory BOS. Lung function and FEV1 stabilized significantly. In summary, ECP offers an attractive corticosteroid-sparing and effective treatment option for advanced intestinal aGvHD and BOS. It is likely that fatal complications related to the prolonged treatment with systemic corticosteroids such as sepsis and resulting multi-organ failure can be reduced.

We assume that this positive treatment effect would even be stronger if ECP is earlier used in the treatment of GvHD and BOS patients. Preferable larger and controlled studies should confirm those results.

## Figures and Tables

**Table 1:** Overview of all patients with acute intestinal GvHD after aHSCT.

Number	Gender	Age	Underlying Disease	Donors’Gender	HLA-Type	Conditioning	GvHD-Prophylaxis	Onset of Intestinal aGvHD (Days)
1	m	59	B-NHL	m	id	2Gy-Flu	CyA/MMF	53
2	f	59	MDS	w	id	Cy-BUS-Flu-ATG	CyA/Mtx	42
3	m	63	mantle cell lymphoma	m	id	2Gy-Flu	CyA/MMF	70
4	m	26	T-NHL	m	id	12Gy-CY-ATG	CyA/Mtx	29
5	m	64	MDS	m	id	BUS-Flu	CyA/Mtx	25
6	m	50	MM	w	id	2Gy-Flu	CyA/MMF	36
7	f	57	MDS	m	dif	BUS-Flu-ATG-Phen	CyA/Mtx	27
8	m	61	MM	m	dif	2Gy-Flu	CyA/MMF	108
9	m	66	CLL	w	id	2Gy-Flu	CyA/MMF	121

(m = male, f = female, B-NHL = B cell Non-Hodgkin’s lymphoma, MDS = Myelodysplastic Syndrome, MM = multiple myeloma, T-NHL = T cell Non-Hodgkin’s lymphoma, CLL = chronische lymphocytic leukemia, id = identical (10/10), dif = different, Gy = Gray, CY = Cyclophosphamid, ATG = Anti-thymocyte globulin, BUS = Busulfan, Flu = Fludarabine, Ams = Amsacrine, Phen = Phenytoin, Cy = Cytarabine, MMF = Mycophenolate-Mofetil, CyA = Cyclosporine A, Mtx = Methotrexate).

**Table 2:** Disease severity of intestinal aGvHD and therapy response.

Grade of aGvHD at Start of ECP	Number of Patients		
	CR	PR	NR
2	0	1	0
3	0	2	2
4	2	0	2

**Table 3:** Overview of all patients with BOS after lung transplantation.

Number	Gender	Age	Underlying Disease	Lung Transplantation Procedure	Immunosuppression after Tx	Acute Tx Rejection	Diagnosis BOS (Year after Tx)
1	m	62	COPD	unilateral	CyA/MMF/Pred	no	4.2
2	m	60	Idiopathic pulmonary fibrosis	bilateral	CyA/MMF/Pred	yes	2.3
3	m	45	Idiopathic pulmonary fibrosis	bilateral	Tac/MMF/Pred	no	0.9
4	f	28	Cystic fibrosis	bilateral	Tac/MMF/Pred	no	4.7
5	m	50	COPD	bilateral	Tac/MMF/Pred	no	1.3
6	m	48	Lung emphysema	bilateral	CyA/MMF/Pred	no	1.4
7	m	51	COPD	bilateral	Tac/MMF/Pred	no	1
8	m	59	Lung emphysema	bilateral	Tac/MMF/Pred	no	2
9	m	56	COPD	bilateral	Tac/MMF/Pred	no no	0.75

(m = male, f = female, Tx = Transplantation, CyA = Cyclosporine A, Tac = Tacrolimus, MMF = Mycophenolate-Mofetil, Pred = Prednisolone).

**Table 4:** Response pattern of BOS patients across patient cohort.

Stage of BOS at Start of ECP	Number of Patients		
	Significant Response to Therapy	Clinical Objectifiable Response but Not Statistically Significant	No Response
1	1	1	0
2	0	1	2
3	1	0	3

**Table 5:** BOS-patient response to ECP and outcome (n.r. = not response, c.r. = clinically objectifiable response but not statistically significant, s.r. = significant response, a = alive, d = dead, Re-LTx = Lung-re-transplantation).

Number	Begin of BOS (Months after LTx)	Stage of BOS at Start of ECP	Begin of ECP (Months after Diagnosis)	ECP Cycles	Treatment Time (in Months)	Treatment Response	Patient Outcome	Cause of Death
1	50	2	9	54	52	n.r.	a	-
2	28	1	0	50	72	s.r.	a	-
3	11	2	3	26	19	c.r.	unknown	-
4	56	2	6	29	30	n.r.	a	-
5	15	3	13	53	28	n.r.	d	Sepsis
6	17	3	2	34	17	n.r.	a (Re-LTx)	-
7	11	3	8	55	31	s.r.	a	-
8	23	3	3	11	5	n.r.	d	Respiratory insufficiency
9	8	1	15	29	14	c.r.	a	-

## Data Availability

Data related to this manuscript is contained within the article or Supplementary Material.

## Supplementary Materials

The following supporting information can be downloaded at: https://www.mdpi.com/article/10.3390/biomedicines10081887/s1. Table S1: Clinical outcome patients with aGvHD (NR = no significant response, PR = partial response, CR = complete response, a = alive, d = dead).
