# Supplementary material for: Effective Extracorporeal Photopheresis of Patients with Transplantation Induced Acute Intestinal GvHD and Bronchiolitis Obliterans Syndrome
Source: Biomedicines. 2022 Aug 4;10(8):1887. doi: 10.3390/biomedicines10081887 (PMC9405770; doi:10.3390/biomedicines10081887)
Supplement: Supplementary file 1 [file biomedicines-10-01887-s001.zip › biomedicines-1838095-supplementary.pdf]

Table S1. Clinical outcome patients with aGvHD

(NR = no significant response, PR = partial response, CR = complete response, a = alive, d = dead)

**Patient number**	**Onset intestinal aGvHD (days after aHSZT)**	**Grade intestinal aGvHD at beginning of ECP**	**Begin of ECP after diagnosis of aGvHD**	**ECP-cycles**	**Treatment duration in days**	**Treatment response**	**Begin response to ECP (in days)**		**Long term patient outcome**	**Cause of death**
							parital	complete		
1	53	3	36	9	29	NR	-	-	d	encephalopathy DD Sepsis
2	42	3	19	8	24	PR	20	-	d	Sepsis
3	70	3	15	6	21	NR	-	-	d	Acute toxic liver failure
4	29	4	40	37	140	NR	-	-	d	Sepsis
5	25	2	62	25	98	PR	66	-	d	Sepsis
6	36	4	21	26	128	CR	32	92	d	Sepsis
7	27	4	8	34	283	CR	35	78	a	-
8	108	4	13	9	32	PR	12	-	d	Sepsis
9	121	4	42	17	80	NR	-	-	d	Sepsis
